# Supplementary material for: Social foraging in vampire bats is predicted by long-term cooperative relationships
Source: PLoS Biol. 2021 Sep 23;19(9):e3001366. doi: 10.1371/journal.pbio.3001366 (PMC8460024; doi:10.1371/journal.pbio.3001366)
Supplement: S1 Text — (DOCX) [file pbio.3001366.s001.docx]

**Supporting Information**

**Fig A: Schematic map of the study site at Tolé, Panama.**

**Fig B. Encounters inside the roost were much longer in duration than encounters outside the roost.**

**Fig C. Differences in social foraging by dyad type.**

**Fig D. Foraging degree centrality is predicted by roosting degree centrality.**

**Fig E. Spectrograms of social calls of common vampire bats inside a roost.**

**Fig F. Variation of call types in multidimensional space.**

**Table A.**  **Within-day effects of roost association rates on foraging encounter rates paired for each daytime period and subsequent night.**

**Table B. No evidence that captive co-feeding rates correlate with social grooming, food sharing, or social foraging time in the wild.**

**Table C: Summary of behavioral context for social calls produced by vampire bats during foraging.**

**Table D. Mean social call parameters.**

**Supplementary methods**

***Subjects: additional details***

The group of previously captive bats was captured outside the hollow tree near Tolé, Panama, then housed in a flight cage at the Smithsonian Tropical Research Institute in Gamboa, Panama from December 14, 2015 to September 19, 2017. Bats were marked with subcutaneous passive integrated transponders (Trovan Ltd. USA) and a visually unique combination of forearm bands (Porzana, National Tag, and birdbands.com). To feed the bats, we provided refrigerated or thawed cattle or pig blood defibrinated with sodium citrate and citric acid. To induce food sharing and grooming among the bats, we conducted 533 fasting trials from March 14, 2016 to September 3, 2017. During each fasting trial, a bat that was isolated without food for a day and night was reintroduced to the group, then observed for 1 hour. Bats were fasted 12 to 23 times (mean = 19.4 fasting trials per bat). For further details see [1].

The 27 wild-caught bats were mist-netted on September 19, 2017. Before sunset, we set up mist-nets in front of the roost entrance to catch exiting bats. Researchers stayed near the nets and removed bats from the mist-net immediately. Bats were kept in cotton cloth bags until we recorded sex, age and reproductive state. We fitted 27 adult females that were not visibly pregnant with proximity sensors.

***Kinship: additional details***

Kinship estimates were based on genotypes from 17 polymorphic microsatellite loci (markers Dr2-2, Dr6-6, Dr7-7, Dr9-9, Dr11-11, Dr15-15, Dr16-16, Dr18-18, Dr30-dr15, Dr31-dr17 [2, Table S2], and markers L0206, L0458, L1154, L2117, L3524, L4050, L4216 [3]). For the first set of markers we used a LI–COR Biosciences DNA Analyser 4300 and the SAGA GT allele scoring software and for the second set of markers we used a SeqStudio Genetic Analyzer and the software Genemapper 6 to amplify and to genotype the sequences. Allele frequencies of the first marker set were based on 91 adult bats from Tolé, Panama, and 42 for the second marker set. All 17 loci passed tests for Hardy-Weinberg Equilibrium (using the R library ‘genetics’) and Linkage Disequilibrium (using the web version of Genepop 4.7.5; markov-chain parameters: 10,000 dememorizations, 1,000 batches, 10,000 iterations per batch). Genotypes were 99.3% complete. We chose the Wang estimator after comparing the performance of all estimators available in the package using the *simrel* function.

***Tracking of encounters among tagged individuals***

We glued custom-made proximity sensors to the bats’ dorsal fur using skin-bonding latex adhesive (Perma-Type surgical bond). Tag weights were in accordance with recommendations for short-term tracking of bats (< 10 % of the body mass [4]). We released the control group back into their roost between 4:50 am to 6:30 am on September 20th. We released the 23 previously captive bats back into the same tree at 8:12 pm.

The signal broadcasted by each proximity sensor (every 2 s) wakes every proximity sensor within 5 - 10 m from ‘sleep mode’, and initiates dyadic encounters between the sender and all receivers. As long as a dyad remains within reception range, the encounter duration and the maximum received signal strength indicator (RSSI) are updated every two seconds. When no signal is received from an encountered partner for 10 s (five times the sampling rate), the encounter is terminated and stored to on-board memory along with the IDs of the partners, a timestamp of the start of the encounter, encounter duration, and the maximum RSSI (as a proxy of the distance between the two partners).

***Identifying foraging bouts and foraging meetings***

We identified a foraging bout of a tagged bat based on the following events: (i) a sudden drop in meeting partners, (ii) an interruption of communication among the proximity sensor and the base station inside the roost, and (iii) base stations on the cattle pasture picking up the signal of the proximity sensor if the bats fly within communication range. A bat which is returning to the roost from a foraging bout should experience a sudden increase in meeting partners and establish communication to the roost base station. We therefore used the meeting architecture of every tagged bat and contact to base stations inside and outside the roost to identify clear individual foraging bouts. We used the custom-made software meeting-splitter [5], which first quantifies the number of simultaneous meeting partners for every second of the study period and then identifies situations where this number falls below a predefined number (in the present case four). We considered such events as potential foraging bouts that we further assessed visually. We confirmed potential bouts by a coinciding interrupt of the communication to the base station inside the roost that indicates absence. In addition, we checked whether the bout was flanked by or contained presence signals at base stations outside the roost confirming presence on the cattle pasture. The latter was not always true since bats may have departed from the roost without coming within communication range of base stations on the pasture and because not all base stations have been operated during all nights. This information merely presents an additional, optional layer of information. We then queried all meetings from the meeting database, which originated during the verified bouts (i.e., ‘foraging encounters’). As a final quality check we verified that both participants of a foraging meeting have currently been on potential foraging bout or have at least not been identified inside the roost. Although it is possible that not all foraging bouts were identified, all foraging encounters were visually verified to have occurred outside the roost.

***Recording video and audio of interactions among foraging vampire bats***

On the night of June 25th, 2019, we took simultaneous and audio and video recordings of free-ranging vampire bats during foraging. The video camera, the ultrasound recorder, and the heavy-duty infrared spotlight that was powered by a 12V car battery were mounted to a tripod in a way that all three devices were facing the same direction. A Dell Latitutde E7450 PC and the car battery were carried inside a backpack. We made recordings at a distance of approximately 3-10 m and using the software Avisoft RECORDER. Gain levels were dynamically adjusted to avoid signal clipping while recording since the volume of vocalizations was highly variable depending on the distance to the recorded animals and the direction the bats were facing while emitting vocalizations. Whenever bats were spotted circling around or landing on a cow, the observer started to record video to match the ultrasonic recordings. We then used the timestamps of the synchronized audio and video to pair the two types of recordings.

In addition, we made sound recordings inside a vampire bat roost only a few hundred meters from the site where we recorded foraging bats. We used the same acoustic recording devices but sampling rate was set to 250 kHz (16-bit depth resolution). Bats were recorded at a distance of ca. 0.5-3 m and gain levels were again adjusted dynamically to avoid signal clipping.

For the acoustic analysis, we determined start and end of each call manually based on the oscillogram and used the Avisoft SASLab’s automatic measurement function to extract spectrum-based parameters. Calls were multi-harmonic, and we took measurements of the fundamental frequency (first harmonic). For one call type (n-shaped calls, see results), the fundamental frequency was very faint, so we measured frequencies from the second harmonic and divided the spectrum-based measurements by 2 to obtain values for the fundamental frequency.

***Study site***


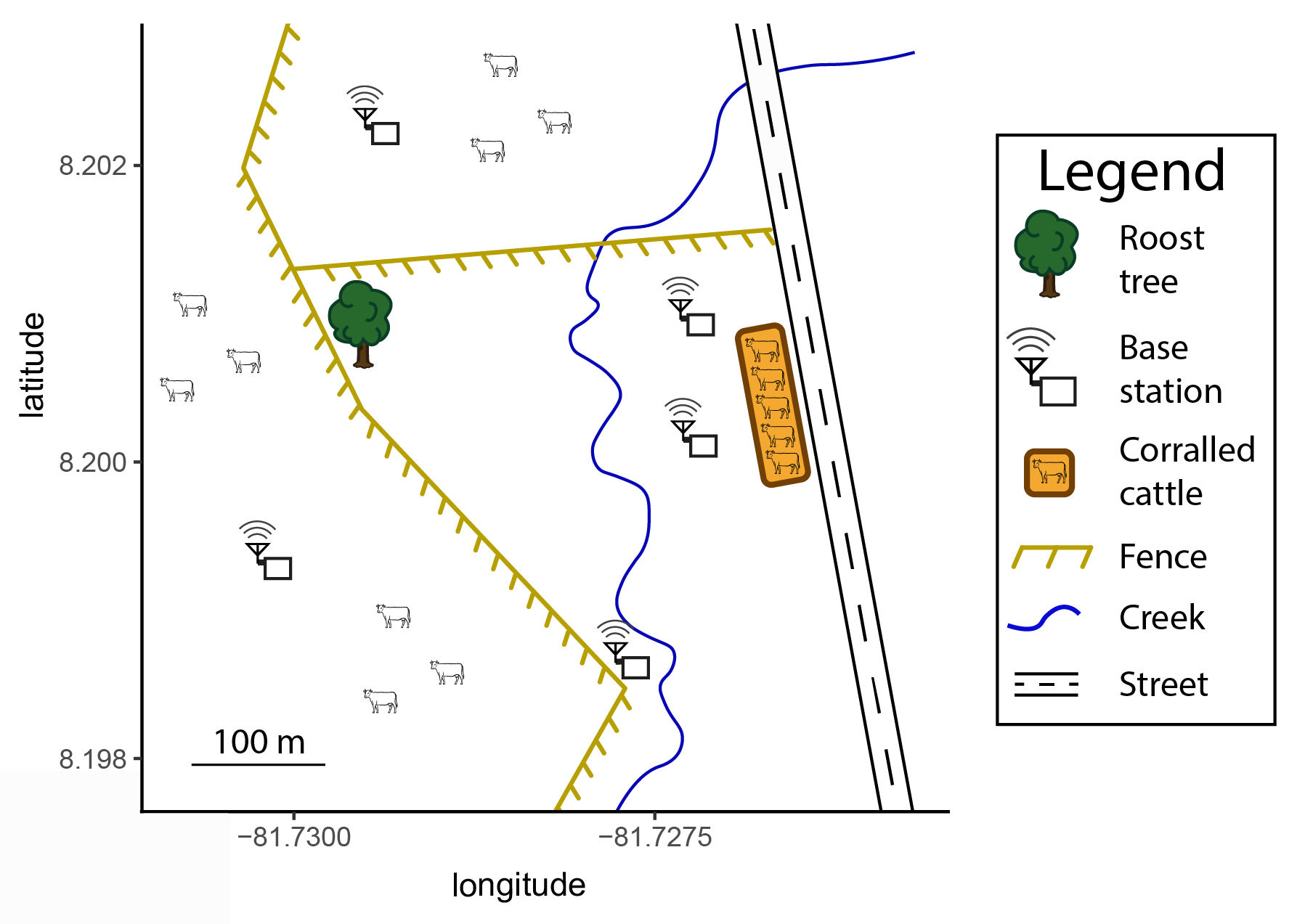


**Fig A: Schematic map of the study site at Tolé, Panama.** Base stations could detect flying bats. Note that the corralled cattle were moving freely starting on day 6 of the study. The pastures north and west of the roost had about 1,500 heads of freely moving cattle. Line drawing of cattle by Imran Razik.

**Supplementary results (supporting figures & tables)**

All data and R code is available on Figshare [6].

**
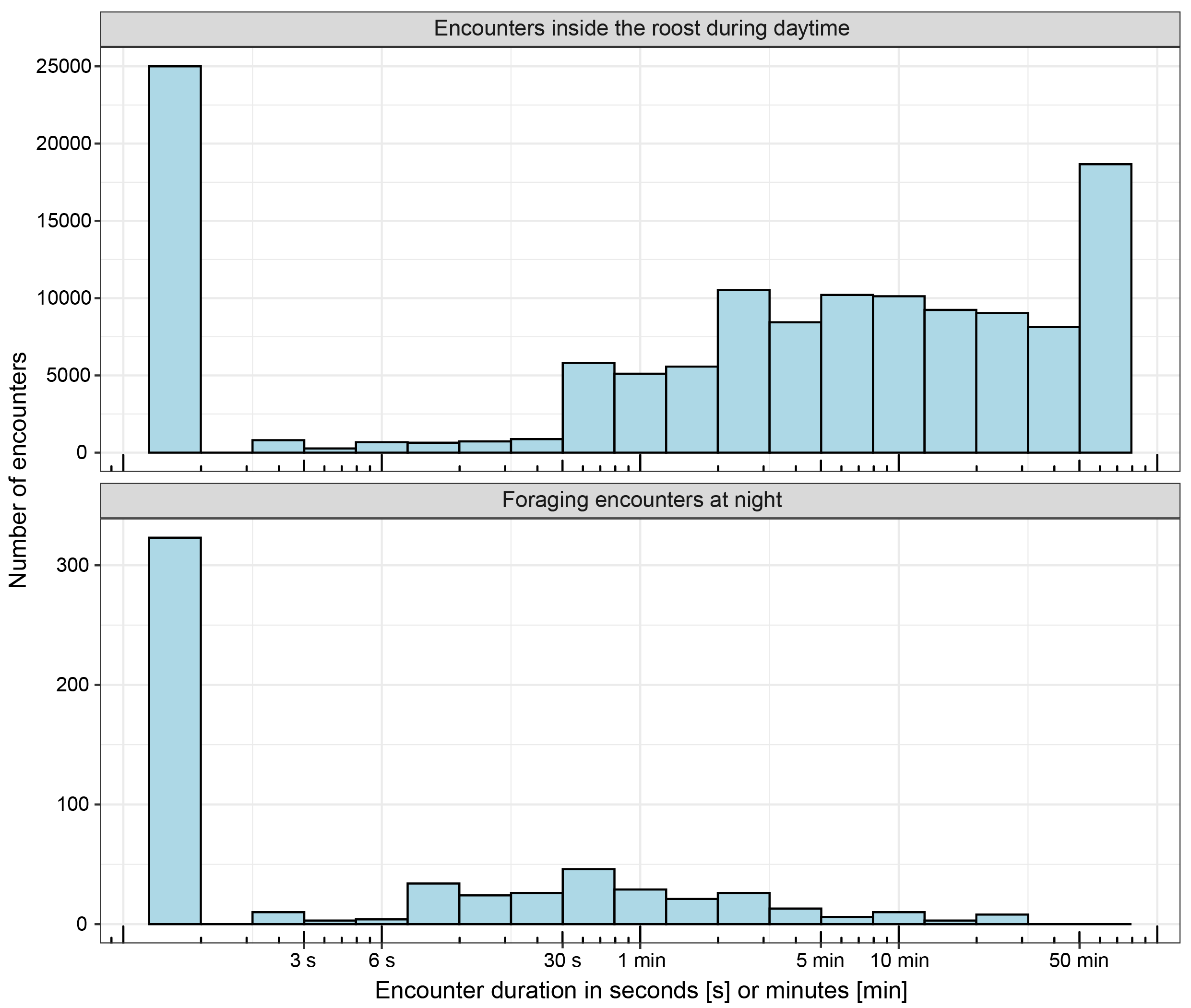
**

**Fig B. Encounters inside the roost were much longer in duration than encounters outside the roost.** Encounters longer than 30 minutes were only observed inside the roost. Histograms show duration of encounters inside the roost during day (top) and foraging encounters at night (bottom). Note that time on the x-axis is plotted on a logarithmic scale. The high bar of short duration is one second, the shortest possible encounter duration (when bats come only briefly within sensor communication range), and the maximum duration was one hour because all encounters were divided at the hour-mark. The underlying data are available in S2 Data.


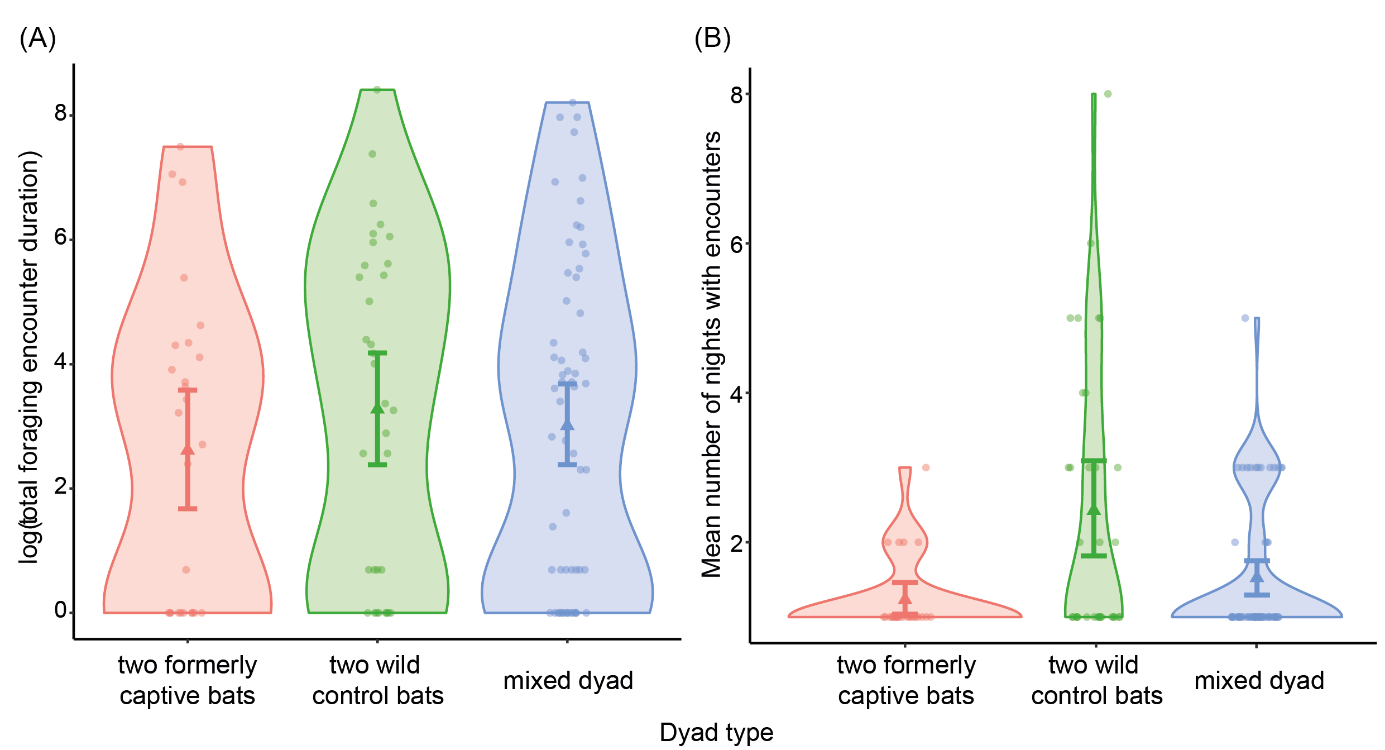


**Fig C. Differences in social foraging by dyad type.** Violin plots show that (A) different types of dyads did not greatly differ in total foraging encounter duration (means (triangles) and bootstrapped 95% CI (error bars) for pairs of two previously captive bats = 2.61 [1.68-3.58], two wild control bats = 3.27 [2.39-4.15], and one previously captive and one wild control bat (mixed dyad) = 3.01 [2.37-3.67]), and that (B) pairs of two wild control bats had more nights with foraging encounters (two previously captive bats = 1.23 [1.07-1.42], two wild control bats = 2.42 [1.82-3.09], mixed dyads = 1.52 [1.31-1.77]). The underlying data are available in S3 Data.


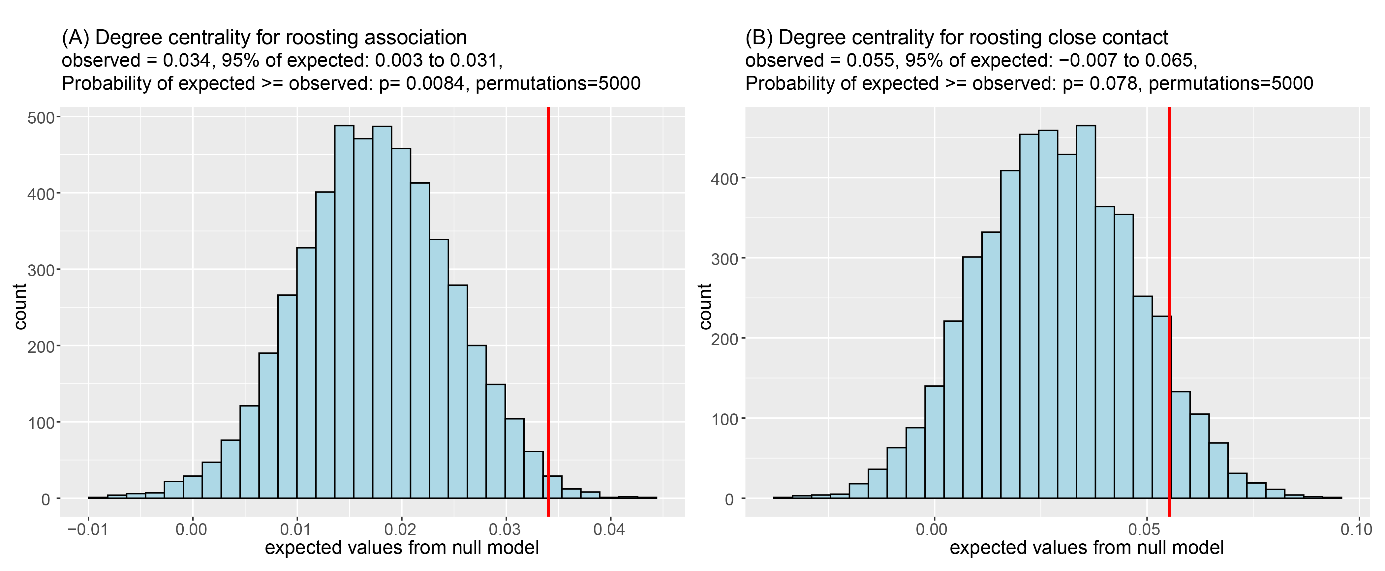


**Fig D. Foraging degree centrality is predicted by roosting degree centrality.** Blue histograms show expected coefficients for the same model fit to randomized data generated by our null model. Red lines show the observed coefficients. Subtitles provide the observed coefficient, the 95% quantiles for the expected coefficients, the one-sided p-value, and number of permutations. Proximity of about 50 cm is required for “roosting associations” and about 2 cm proximity is needed for “close contact”, so close-contact networks are sparser and based on fewer observations. The script underlying this permutation test is available as S4 Data.


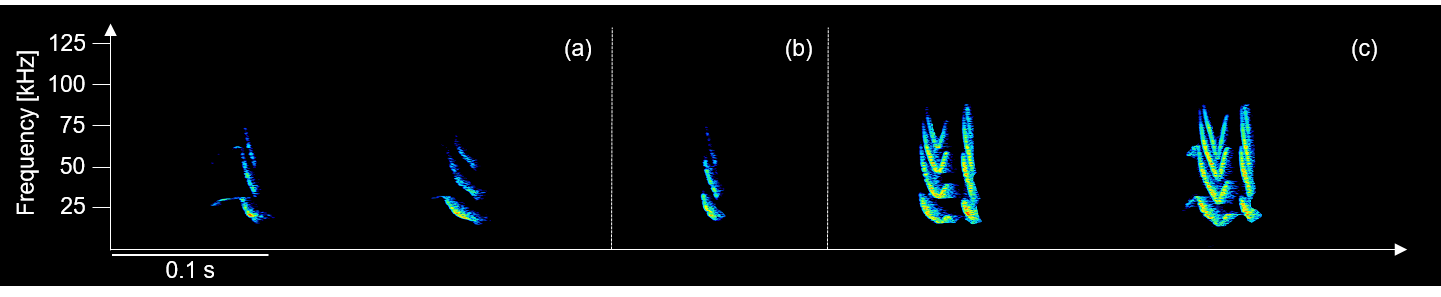


**Fig E. Spectrograms of social calls of common vampire bats inside a roost.** Tens of bats present. Call types were (a) “undulated down sweep”, (b) “down sweep”, and (c) “u-shaped calls followed by down sweep”. Inter-call intervals have been modified for the figure.


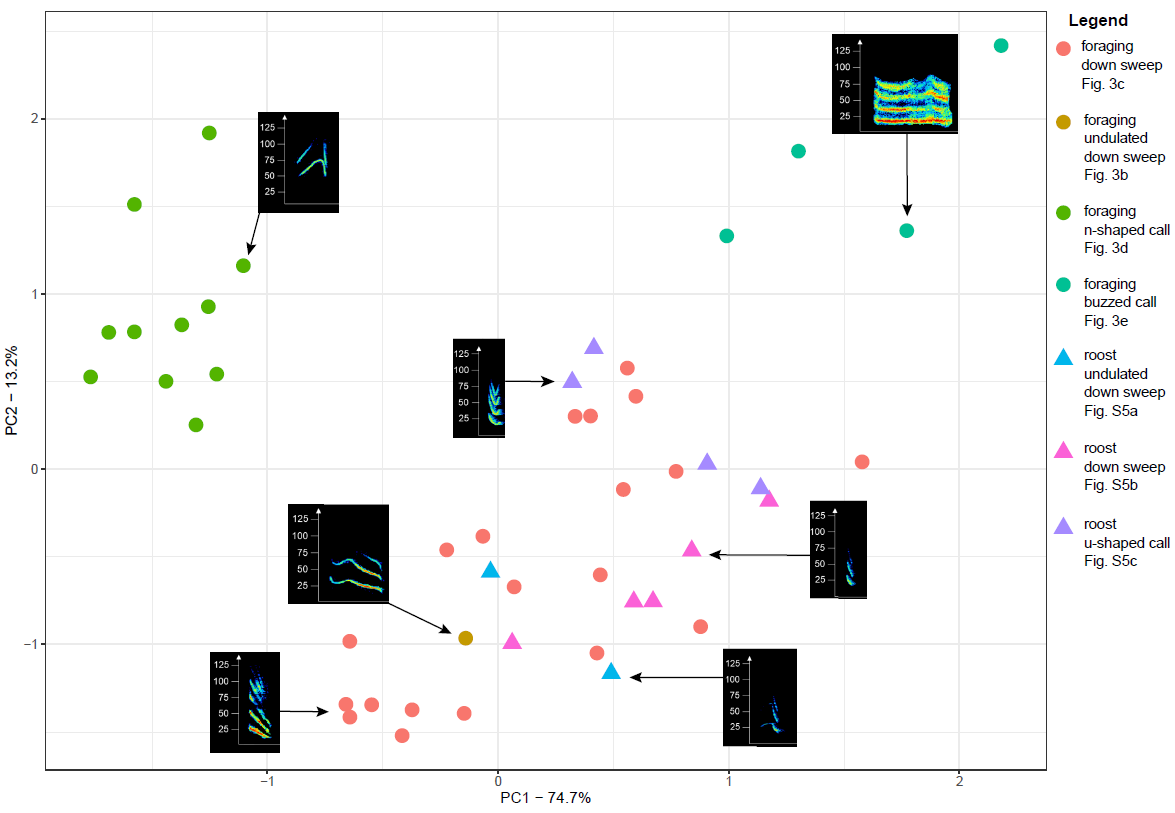


**Fig F. Variation of call types in multidimensional space.** X and Y axis are first two principal components from spectrum-based parameters measured at 11 positions across each call. Buzz calls produced in antagonistic context and the n-shaped calls were produced during foraging and were distinct from the remaining call types in multivariate space. The legend refers to spectrograms of the calls in Fig 3 & Figure E in S1 Text. See Table D in S1 Text for call parameter measurements. The underlying data for creating this figure are available in S5 Data.

**Table A.**  **Within-day effects of roost association rates on foraging encounter rates paired for each daytime period and subsequent night.** Data from day nine was lost due to equipment malfunction. Evidence for a within-day effect was only detected for day 4, but the model beta coefficients across the 9 days was overall biased above zero (as shown by nonparametric bootstrapping).

| day/night | association | Beta statistic | | p | N | |
| --- | --- | --- | --- | --- | --- | --- |
| 2 | 50 cm | -0.001 | 0.98 | | 46 | |
|  | 2cm | 0.016 | 0.57 | | 46 | |
| 3 | 50 cm | 0.035 | 0.13 | | 48 | |
|  | 2cm | 0.010 | 0.68 | | 48 | |
| 4 | 50 cm | 0.098 | 0.001* | | 46 | |
|  | 2cm | 0.084 | 0.013* | | 45 | |
| 5 | 50 cm | 0.047 | 0.11 | | 45 | |
|  | 2cm | 0.001 | 0.96 | | 44 | |
| 6 | 50 cm | 0.027 | 0.28 | | 41 | |
|  | 2cm | 0.003 | 0.91 | | 40 | |
| 7 | 50 cm | 0.030 | 0.36 | | 39 | |
|  | 2cm | 0.032 | 0.35 | | 39 | |
| 8 | 50 cm | -0.021 | 0.33 | | 38 | |
|  | 2cm | -0.009 | 0.66 | | 37 | |
| 10 | 50 cm | -0.007 | 0.64 | | 37 | |
|  | 2 cm | 0.009 | 0.64 | | 34 | |
| Mean | 50 cm | 0.026 [0.004 to 0.051] | | |  |  |
| [95% CI] | 2 cm | 0.018 [0.003 to 0.04] | | |  |  |

**Table B. No evidence that captive co-feeding rates correlate with social grooming, food sharing, or social foraging time in the wild.** Rows show correlations with captive co-feeding rates.

| Measure correlated with captive co-feeding rate | N bats | Mantel test, Pearson’s r | Mantel test, p-value | Double permutation test, Pearson’s r | Double permutation test p-value |
| --- | --- | --- | --- | --- | --- |
| Social grooming | 31 | 0.006 | 0.37 | 0.008 | 0.36 |
| Food sharing | 31 | 0.03 | 0.20 | 0.015 | 0.28 |
| Foraging encounter rate | 20 | 0.02 | 0.30 | 0.003 | 0.42 |

**Table C: Summary of behavioral context for social calls produced by vampire bats during foraging.** n = n-shaped call, ds = down sweep, z = buzz call, uds = undulated down sweep; consecutive calls were recorded within less than a second (-) or more than one second (---). Since the recordings were obtained from freely moving animals, some calls were very faint and call sequences may therefore miss individual calls.

|  |  |  | **Number calls per call type** | | | |  |
| --- | --- | --- | --- | --- | --- | --- | --- |
| **Behavioral context bats** | **Behavioral context cows** | **Position of vocalizing bat** | **n** | **ds** | **z** | **uds** | **Call sequence** |
| two bats feeding on one cow | cow moving head, scratching itself | on cow for first two calls then invisible | 0 | 6 | 0 | 0 | ds---ds---ds---ds---ds-ds |
| one bat approaching cow in flight, second bat flies up behind cow; bats circle in flight and one bat lands on cow | grazing calmly | unclear | 0 | 0 | 0 | 2 | uds-uds |
| 1 bat on cow, 2nd bat circles around flying, lands and takes off immediately | grazing calmly | on cow | 3 | 1 | 0 | 0 | n-n-n-ds |
| 1 bat on cow, 1 flying | grazing calmly | unclear | 1 | 0 | 0 | 0 | n |
| 3 bats feeding on 3 cows within few meters (see S1 Video) | grazing calmly | all three bats on cows vocalize sporadically | 10 | 0 | 0 | 0 | n---n---n-n-n---n---n---n---n---n |
| 3 bats on 3 cows, 1 bat flying by | grazing calmly | Unclear | 1 | 1 | 0 | 0 | ds-n |
| 1 bat on 1 cow | grazing calmly | Unclear | 1 | 0 | 0 | 0 | n |
| 1 bat feeding on cow, second bat flies by, lands, both engage in fight and fly away (see S2 Video) | grazing calmly | on cow | 3 | 2 | 3 | 0 | n-n-n-ds-z-z-z---ds |
| 1 bat flying | grazing calmly | Unclear | 0 | 3 | 0 | 0 | ds---ds-ds |
| 2 bats feeding on 1 cow | grazing calmly | on cow | 5 | 0 | 0 | 0 | n---n---n-n-n |
| 2 bats feeding on 1 cow | cow moves ear disturbing 1 feeding bat | disturbed bat on cow | 3 | 1 | 0 | 0 | n-ds-n-n |
| 2 bats feeding on 1 cow; 1 bat drinking, second bat moving around, making body contact with first bat, gets hit by the ear of the cow, then both bats start pushing each other from one side of the cow neck to the other side (see S3 Video) | grazing calmly; slaps a bat with ear | on cow | 5 | 8 | 1 | 0 | n-ds---n-n-n---ds-ds-ds---ds---ds-ds-n-ds-z |
| same 2 bats feeding on same cow (separate wounds) | grazing calmly, walking slowly | on cow | 6 | 2 | 0 | 0 | n-ds-n---n---n-n-n-ds |
| 2 bats on one cow; 1 flies off, returns, finally both fly off (see S4 Video) | cow is running | bats on cow | 4 | 3 | 0 | 0 | n-n-n---n-n---n-ds-ds-ds |

**Table D. Mean social call parameters.** Parameters include call duration, peak frequency of maximum amplitude, minimum fundamental frequency, and maximum fundamental frequency for call types recorded during foraging and inside the roost. The standard deviation is given in parentheses.

| Context | Call type | n | Call  duration | Peak  frequency | Minimum  frequency | Maximum  frequency |
| --- | --- | --- | --- | --- | --- | --- |
| Foraging | Down sweep | 20 | 4.19 (2.25) | 24.37 (3.93) | 19.64 (3.37) | 33.14 (3.59) |
|  | Undulated down sweep | 1 | 20.09 | 23.40 | 17.50 | 34.10 |
|  | n-shaped call | 11 | 8.99 (1.42) | 32.87 (2.09) | 27.23 (1.30) | 36.41 (1.33) |
|  | Buzz call | 4 | 13.21 (11.68) | 16.80 (3.26) | 13.15 (3.06) | 22.43 (1.79) |
| Roost | Undulated down sweep | 2 | 28.15 (3.61) | 19.00 (0.99) | 17.05 (1.06) | 31.35 (0.49) |
|  | u-shaped call | 4 | 20.88 (4.73) | 22.23 (2.36) | 18.00 (2.25) | 32.48 (1.11) |
|  | Down sweep | 5 | 11.32 (2.13) | 20.44 (1.82) | 17.38 (2.26) | 32.38 (2.11) |

**Reference**

1. Carter GG, Farine DR, Crisp RJ, Vrtilek JK, Ripperger SP, Page RA. Development of new food-sharing relationships in vampire bats. Curr Biol. 2020;30(7):1275-9. e3.

2. Ripperger SP, Carter GG, Duda N, Koelpin A, Cassens B, Kapitza R, et al. Vampire bats that cooperate in the lab maintain their social networks in the wild. Curr Biol. 2019;29(23):4139-44. e4.

3. Ripperger S, Waurick I, Blom M. Tri- and tetranucleotide microsatellite markers developed based on the genome of the common vampire bat (*Desmodus rotundus*). Museum für Naturkunde Berlin (MfN) - Leibniz Institute for Evolution and Biodiversity Science; 2021. Doi: 10.7479/e1hc-ff78.

4. Amelon SK, Dalton DC, Millspaugh JJ, Wolf SA. Radiotelemetry techniques and analysis. In: Kunz TH, Parsons S, editors. Ecological and behavioral methods for the study of bats. 2 ed. Baltimore, Maryland: The John Hopkins University Press; 2009. p. 57-77.

5. Ripperger S. Meeting Splitter. Museum für Naturkunde Berlin (MfN) - Leibniz Institute for Evolution and Biodiversity Science; 2019. Doi: 10.7479/ytdf-wf05.

6. Ripperger S, Carter G. Data and R code for "Social foraging in vampire bats is predicted by long-term cooperative relationships"2021. Doi: 10.6084/m9.figshare.14529588.v2.
