# Supplementary material for: Social foraging in vampire bats is predicted by long-term cooperative relationships
Source: PLoS Biol. 2021 Sep 23;19(9):e3001366. doi: 10.1371/journal.pbio.3001366 (PMC8460024; doi:10.1371/journal.pbio.3001366)

Time of foraging bouts by bat and day (see S6 Data for the underlying data)
Each row shows a bat on a specific date (20–29). Points are encounters within bouts.

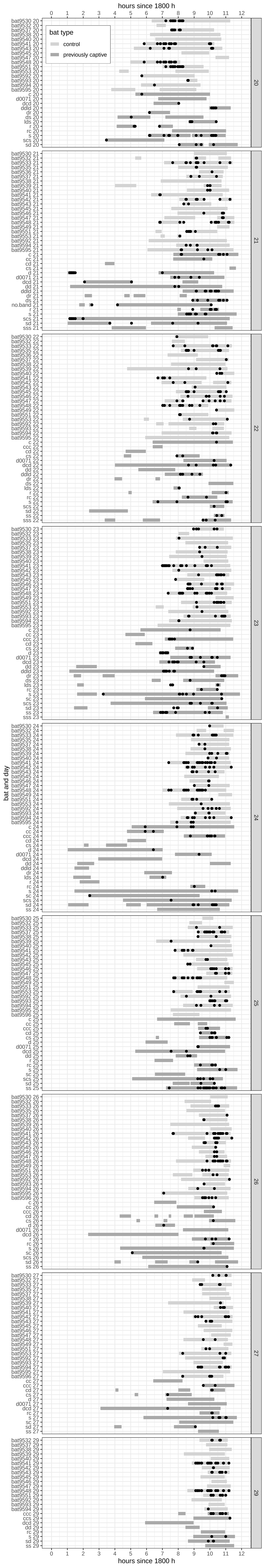

Supplement: S1 Fig — (PDF) [file pbio.3001366.s002.pdf]
