# Supplementary material for: Social foraging in vampire bats is predicted by long-term cooperative relationships
Source: PLoS Biol. 2021 Sep 23;19(9):e3001366. doi: 10.1371/journal.pbio.3001366 (PMC8460024; doi:10.1371/journal.pbio.3001366)
Supplement: Attachment — Submitted filename: response_to_reviewers03.docx [file pbio.3001366.s014.docx]

Response to reviewers

**We thank the reviewers for their constructive comments, which were thoughtful, very helpful, and greatly improved the manuscript. Below, you’ll find our responses to each point raised in bold. We hope that our manuscript is now suitable for publication in PLOS Biology.**

**--Simon Ripperger and Gerry Carter**

Dear Simon,

Thank you very much for submitting your manuscript "Social foraging in vampire bats is predicted by long-term cooperative relationships" for consideration as a Short Report by PLOS Biology. As with all papers reviewed by the journal, yours was evaluated by the PLOS Biology editors as well as by an Academic Editor with relevant expertise and by three independent reviewers.

You'll see that the reviewers are broadly positive about your study, but each has a number of textual and/or presentational requests to improve the paper (plus some minor analyses). Based on the reviews, we will probably accept this manuscript for publication, provided you satisfactorily address the remaining points raised by the reviewers. Please also make sure to address the following data and other policy-related requests.

IMPORTANT:

a) Please attend to all of the requests from the reviewers. We note reviewer #2's suggestion that you remove the section (line 405) about calls; we actually think that this would be of interest to our readers, and if you decide to retain it, we suggest that you move one of the relevant (and rather interesting) supplementary Figs into the main main manuscript. Indeed, as a Short Report, you are allowed up to 4 main Figures, so if you feel that one of the other supplementary Figures would help address any of the clarity issues raised (especially for our broader readership, then do feel free to "promote" it to the main paper.

**We agree and would like to retain this section. As suggested, we have moved** **the supplementary figure showing the variation in calls recorded from foraging bats into the main text. We have also annotated this figure to make it easier to interpret. See below.**

**Figure 3. Spectrograms of social calls of common vampire bats flying near or attacking free-ranging cattle on a pasture.** Behavioral context was derived from synchronized video, and we identified the calling bat when mouth movements were visible. Calls include (a) echolocation calls (biosonar), (b) undulated downsweep, which was only observed in one recording where two bats where flying near a cow, (c) downsweep calls, (d) n-shaped calls, and (e) buzz calls recorded while two bats engaged in antagonistic behavior on a single cow. Inter-call intervals were modified for the figure, except for the call sequence in panel (e), a sequence recorded from two aggressively interacting bats.

b) Please address my Data Policy requests further down. Essentially, we need the directly underlying data for some of the Figs to be provided, and for the location of the data to be cited in the legends.

**In addition to the full R script and data on Figshare, we also now provide a separate data file for each Figure as supplementary material.**

As you address these items, please take this last chance to review your reference list to ensure that it is complete and correct. If you have cited papers that have been retracted, please include the rationale for doing so in the manuscript text, or remove these references and replace them with relevant current references. Any changes to the reference list should be mentioned in the cover letter that accompanies your revised manuscript.

We expect to receive your revised manuscript within two weeks.

To submit your revision, please go to <https://www.editorialmanager.com/pbiology/> and log in as an Author. Click the link labelled 'Submissions Needing Revision' to find your submission record. Your revised submission must include the following:
-  a cover letter that should detail your responses to any editorial requests, if applicable, and whether changes have been made to the reference list
-  a Response to Reviewers file that provides a detailed response to the reviewers' comments (if applicable)
-  a track-changes file indicating any changes that you have made to the manuscript. 

NOTE: If Supporting Information files are included with your article, note that these are not copyedited and will be published as they are submitted. Please ensure that these files are legible and of high quality (at least 300 dpi) in an easily accessible file format. For this reason, please be aware that any references listed in an SI file will not be indexed. For more information, see our Supporting Information guidelines:
<https://journals.plos.org/plosbiology/s/supporting-information>  

*Published Peer Review History*
Please note that you may have the opportunity to make the peer review history publicly available. The record will include editor decision letters (with reviews) and your responses to reviewer comments. If eligible, we will contact you to opt in or out. Please see here for more details:
<https://blogs.plos.org/plos/2019/05/plos-journals-now-open-for-published-peer-review/>

*Early Version*
Please note that an uncorrected proof of your manuscript will be published online ahead of the final version, unless you opted out when submitting your manuscript. If, for any reason, you do not want an earlier version of your manuscript published online, uncheck the box. Should you, your institution's press office or the journal office choose to press release your paper, you will automatically be opted out of early publication. We ask that you notify us as soon as possible if you or your institution is planning to press release the article.

*Protocols deposition*
To enhance the reproducibility of your results, we recommend that if applicable you deposit your laboratory protocols in [protocols.io](http://protocols.io/), where a protocol can be assigned its own identifier (DOI) such that it can be cited independently in the future. Additionally, PLOS ONE offers an option for publishing peer-reviewed Lab Protocol articles, which describe protocols hosted on [protocols.io](http://protocols.io/). Read more information on sharing protocols at <https://plos.org/protocols?utm_medium=editorial-email&utm_source=authorletters&utm_campaign=protocols>

Please do not hesitate to contact me should you have any questions.

Sincerely,

Roli


Roland G Roberts, PhD,
Senior Editor,
[rroberts@plos.org](mailto:rroberts@plos.org),
PLOS Biology

------------------------------------------------------------------------
DATA POLICY:

You may be aware of the PLOS Data Policy, which requires that all data be made available without restriction: <http://journals.plos.org/plosbiology/s/data-availability>. For more information, please also see this editorial: <http://dx.doi.org/10.1371/journal.pbio.1001797> 

We note that you present raw data and R scripts in the Figshare deposition. However, we also require the numerical values that underlie the figures and results of your paper be made available in one of the following forms:

1) Supplementary files (e.g., excel). Please ensure that all data files are uploaded as 'Supporting Information' and are invariably referred to (in the manuscript, figure legends, and the Description field when uploading your files) using the following format verbatim: S1 Data, S2 Data, etc. Multiple panels of a single or even several figures can be included as multiple sheets in one excel file that is saved using exactly the following convention: S1_Data.xlsx (using an underscore).

2) Deposition in a publicly available repository. Please also provide the accession code or a reviewer link so that we may view your data before publication. 

Regardless of the method selected, please ensure that you provide the individual numerical values that underlie the summary data displayed in the following figure panels as they are essential for readers to assess your analysis and to reproduce it: Figs S2, S3, S4, S7. NOTE: the numerical data provided should include all replicates AND the way in which the plotted mean and errors were derived (it should not present only the mean/average values).

IMPORTANT: Please also ensure that figure legends in your manuscript include information on where the underlying data can be found, and ensure your supplemental data file/s has a legend.

**For all figures mentioned and for ESM 2, we have uploaded .csv or .xlsx files as supplementary files and we also included this information in the figure legends.**

Please ensure that your Data Statement in the submission system accurately describes where your data can be found.

**We now provide the link to the Figshare site that contains the raw data and R scripts for all analyses.**

------------------------------------------------------------------------
DATA NOT SHOWN?

- Please note that per journal policy, we do not allow the mention of "data not shown", "personal communication", "manuscript in preparation" or other references to data that is not publicly available or contained within this manuscript. Please either remove mention of these data or provide figures presenting the results and the data underlying the figure(s).

------------------------------------------------------------------------

REVIEWERS' COMMENTS:


Reviewer #1:

This paper is interesting, particularly in the use of proximity sensors in free-ranging bats. Overall, the data appears sound and the writing clear.

**Thank you!**

Specific comments on the manuscript:
Line 89:  change to "departing, following, or foraging"

**Fixed**

Lines 101-102:  state which species of vampire bat

**Fixed**

Lines 139 and 141:  If you recorded 277 co-feeding cases, why were only 201 use in the analyses?

**We have clarified that not all individuals during all events could be identified. Revised text reads: “… 277 cases of co-feeding where two bats were observed feeding from the same blood spout at the same time (from 1050 h of observation from 70 nights). In 201 of these cases, both bats were clearly identified.”**

Line 157:  Can you give more details about mechanism behind the proximity sensors, or at least reference the supplemental material here.

**In accordance with a comment of reviewer 2 (see below), we now reference the supplement and another key reference (Ripperger et al. 2016) that describes the sensor nodes and in particular the relationship between signal strength and distance in more detail. Revised text now reads:**

**“We placed custom-made proximity sensors on all 50 female common vampire bats (sensor mass: 1.8 g; 4.5-6.9 % of each bat’s mass) that automatically documented dyadic associations among all 50 tagged bats when those come within the reception range of 5-10 m (868/915MHz, see supplement, Ripperger et al., 2019; Ripperger et al., 2020b). To log encounters, each proximity sensor broadcasted a signal every two seconds to update the duration of each encounter. We used 1 s as the duration of encounters that were shorter than two successive signals (i.e., encounters shorter than two seconds). The maximum signal strength of each encounter can be used as an estimate for a minimum proximity between two tagged bats during the encounter by comparing the signal intensity to a calibration curve (Ripperger et al., 2016, Ripperger et al., 2019; Ripperger et al., 2020b).”**

Lines 211-212:  what is meant by "visual light"? Light not visible to the cows? The bats?

**Fixed. Yes we meant infrared light not visible to humans, cows, or bats. Text now reads:**

**“One observer (SPR) moved with a herd of about 20 grazing cattle without visible light, only using the viewfinder of the IR camera.”**

Line 214:  Is this a different roost from the one used for the other data collection? Please clarify.

**Yes, this is correct. Revised text now reads:**

**“At Tolé, we only observed two occasions, where two bats stopped at the cattle pasture and were associated (for 3.5 and 4.6 minutes). When releasing the corralled cattle in the morning we observed bite marks but to avoid changing their behavior, we did not get close enough to the cattle at night to record audio or video of bats interacting. To collect direct observations on foraging behavior, we recorded simultaneous audio and video of bat foraging behavior at a different farm near La Chorrera…**

**…To compare social calls made during foraging with calls from inside a roost, we used the same recording equipment to record social calls from a roost only a few hundred meters from the foraging site at La Chorrera.”**

Line 220:  Subsequently

**Typo fixed.**

Line 252:  What is meant by "hour bin" in this sentence?

**Thanks, that was somewhat unclear. We deleted “bin”. Text now reads:**

**“For instance, if two bats met twice in the same hour, this is still one encounter.”**

Lines 352-354:  Please reword/clarify this sentence. The variation was greater than expected?

**Fixed. Yes, the variation was greater than expected from random foraging.**

**Original wording: “The variation in number of hours in which two bats reunited was greater than expected from our null model…”**

**New wording:** **“Pairs of bats varied in the number of hours in which they reunited, and this variation was greater than expected from our null model…”**

Line 392:  p = 0.063 is not significant... reword this sentence

**We revised the text and it now reads:** “**…foraging encounter time was predicted … by social grooming (QAP, β = 26.5, n = 22, p = 0.032), but the effect of social grooming was unclear when controlling for bout overlap (MRQAP, β = 0.12, p = 0.063).”**

Line 399:  Please explain why this is only "weak" evidence, when your statistical results don't differ from what you consider stronger evidence for other comparisons above.

**We intended to highlight the differences in evidence when testing for social differentiation in captive versus free-ranging bats. In the field, observed social differentiation was 4.36, p < 0.001. But in captivity, the same metric was 2.1, p = 0.047. To make this clearer, we revised the text to read:**

**“In contrast to the evidence for social differentiation in the field, we detected only weak evidence for preferred associations during co-feeding in captivity (social differentiation = 2.10, p = 0.047 when controlling for hour, p = 0.041 when not controlling for hour)”…**

**To make the comparison more obvious, we also now use the term “social differentiation” for the field result, rather than the equivalent wording: “coefficient of variation in association”.**

Line 502:  "not a limited resource" seems to contradict what you say above and below about competition for wounds.

**We agree. The revised text now reads**: **… “blood from an open wound is not as limited of a resource as a discrete food item”…**

End of discussion:  Include a concluding statement at the end.

**Our new revised final sentence is:**

**“Rapid standardized high-throughput methods for measuring social network structure, such as social proximity sensors, allow for social networks to be mapped quickly across multiple populations and species, enabling comparative studies investigating evolutionary and ecological drivers of social complexity across species.”**

Best of luck on your paper.

**We thank the reviewer for these helpful suggestions for improving the clarity of the writing.**

Reviewer #2:

This is an interesting topic, asking pertinent questions in a relevant and fascinating model system. An awful lot of work has gone into gathering these data.

**Thank you! We appreciate these nice words.**

Figure 1 was particularly useful for explaining the framework.

I do have some comments and suggestions.

General comments:

(1) What are the effects of having those animals in captivity? What is the carry-over effect? How natural would they be expected to behave, given they have been fed for a long period of time under captive conditions?

**This is a good question, but we had no clear theoretical or *a priori* predictions about how previous captivity would affect foraging. The possible carry-over effects of previous captivity are best assessed by comparing the behavior of the previously captive bats and control bats. That is, the previously captive bats foraged earlier and less consistently than the control group. We explain these differences in the main text in the section labeled “*Previous captivity influenced departures and foraging”*, but for the sake of brevity we don’t interpret them in great detail, and we allow readers to explore these differences further in the supplement (ESM2).**

(2) What is the accuracy of the proximity loggers?

**The temporal resolution is 2 seconds meaning that all pairwise encounters are updated every two seconds. The spatial accuracy depends on humidity and tag orientation and varies with distance. It is, therefore, important to collect calibration data on-site for every field deployment. The plot below is from Ripperger et al. 2019 Current Biology and the data for this plot have been collected at our field site near Tolé. The X-axis shows the true proximity and the y-axis is signal strength (used as a proxy for distance). It shows the variation in RSSI values for loggers at distances of 2 cm (left) to 5 meters (right) during controlled tests at the field site in Panama. The two red lines show standard thresholds used for defining an encounter or close contact. The proximity sensors are particularly useful to distinguish meetings that originated within close contact since the drop in signal intensity is most pronounced within 0-1 m. We now cite a paper of ours (Ripperger et al. 2016 Ecol Evol) that described the proximity sensors in detail and has a stronger focus on the relationship between signal intensity and the distance between tagged individuals than the cited references.**

**We added this reference to the methods part. We hope that the methodological details that we provide in the main text and the supplement along with the cited methods papers will allow understanding the data collection in detail.**

(3) What is the time between the data was gathered in the published study (Ripperger et al 2019) and it being used in this study? What is the likely stability of these associations? If published in 2019, likely gathered some time before that.

**The grooming and food-sharing data in captivity was collected from March 14, 2016 to September 3, 2017, and the foraging data was collected starting on September 21, 2017. We realize that the timing of data collection in captivity was somewhat unclear in the original version of the manuscript. Thanks for bringing this to our attention. We now provide the exact dates in the supplement. Revised text reads:**

**“The group of previously captive bats was captured outside the hollow tree near Tolé, Panama, then housed in a flight cage at the Smithsonian Tropical Research Institute in Gamboa, Panama from December 14, 2015 to September 19, 2017. Bats were marked with subcutaneous passive integrated transponders (Trovan Ltd. USA) and a visually unique combination of forearm bands (Porzana, National Tag, and birdbands.com). To feed the bats, we provided refrigerated or thawed cattle or pig blood defibrinated with sodium citrate and citric acid. To induce food sharing and grooming among the bats, we conducted 533 fasting trials from March 14, 2016 to September 3, 2017.”**

**To our embarrassment, we also realized we mis-stated the number of months the bats were in captivity. It was 21 months not 22 months. We fixed this throughout the text.**

(4) Mass of loggers. These are high as a % for flying animals. General level of acceptance is 5%. 6.9% is high. Could this have restricted their flight duration? And caused clustering due to reduced flight durations?

**We agree that logger mass is an important factor that requests careful consideration for each biologging study. In our opinion, an acceptable tag mass should be defined in the light of species-specific traits and the goals of the study.**

**Tagging effects are not likely to have an impact on our analyses because the effect of the logger mass (1.8 g) would be the same for the test group and control group, so even if the loggers did affect flight durations, they could not create the social effects we saw (preferred associations between some bats or correlations with captive behavior).**

**However, we also highly doubt that the load of the tags (4.5-6.9% of their pre-fed body weight) seriously burdened the flights of this particular bat species for several reasons.**

**First, the 5% rule was designed for small insect-eating bats that catch their food in flight. A main concern was that attaching sensors may decrease maneuverability and impact the foraging success of aerial-hawking bats. Vampire bats do not need to commute long distances or catch insects in flight. Unlike fruit and insect-eating bats, they can feed on cattle which are near their roost and drink blood while resting on the ground or their animal host.**

**Second, the mass we used for percentage bodyweight was pre-feeding mass, and a lower estimate would be obtained after the bats had fed. Vampire bat body mass varies greatly before and after feeding. Our own data show that bats gain/ lose on average 15% of their body mass depending on when they fed. Other studies report that they can gain 37% -- 100% of their body mass immediately after feeding on blood, and that even after losing weight from peeing, they may have still gained 20-30% of pre-feeding mass. Vampire bats have a number of unique adaptations for taking flight (and probably commuting) after accumulating large amounts of weight.**

**References:**

- **Breidenstein, C. P. (1982). Digestion and assimilation of bovine blood by a vampire bat (Desmodus rotundus). Journal of Mammalogy, 63(3), 482-484.**
- **McFarland W. N.; Wimsatt W. A. (1965). "Urine flow and composition in the vampire bat". Am. Zool. 5: 662–667.**
- **Schutt J. E. A.; Altenbach W. A.; Chang J. S.; Cullinane Y. H.; Hermanson D. M.; Muradali J. W.; Bertram F. (1997). "The dynamics of flight-initiating jumps in the common vampire bat Desmodus rotundus". *Journal of Experimental Biology*. 200 (23): 3003–3012.**
- **Wimsatt, W. A. (1969). Transient behavior, nocturnal activity patterns, and feeding efficiency of vampire bats (Desmodus rotundus) under natural conditions. *Journal of Mammalogy*, *50*(2), 233-244.**

**To give one example from the field rather than the lab, this image below taken by Jon Flanders shows a male vampire bat that looks pregnant because it has just fed. It was captured at emergence and again 3 hours later after feeding. During the capture, it peed several times, but it was still 6 g heavier (about 17% body mass) when it was weighed. The extra blood in its belly, after urination, is the weight of three proximity sensors.**

**Third, female vampire bats are typically either nursing or pregnant (they are reproductive all year round but have a 6-month gestation period). We made sure that none of our subjects were pregnant. A newborn pup is again a significant percentage of its mother’s non-pregnant weight (weighing 6 g at birth). Our tagged test subjects were therefore lighter than many female vampire bats are under natural conditions.**

**Fourth, as stated in the supplement, tag weights were in accordance with some recommendations for short-term tracking of bats (< 10 % of the body mass (Amelon et al., 2009) and our study qualifies as ‘short-term’ since tags dropped off after ~10-14 days).**

Line 184, Lines 233-256:
I struggled to follow everything in this section. Is it possible please to walk the reader a little slower through what was done here.

**We agree. We revised Line 184 as follows:**

**Original: “To log presence and co-occurrence of foraging bats at points outside the roost during the night, we placed base stations (which can detect tagged bats at distances of about 150 m) at the roost and at 5 other locations in the surrounding cattle pastures.”**

**New: “To help localize bats, we used base stations that can detect tagged bats at distances of about 150 m. We placed these stations at the roost and at 5 other locations in the surrounding cattle pastures to help localize individuals and encounters as inside or outside the roost.”**

**We also rewrote lines 233- 256. Revised text now reads:**

**“*Statistical analysis of foraging behavior***

**To estimate foraging bouts, we calculated the periods when each bat was distant from the roost tree (ESM File 2). Then, to test whether the previously captive bats and never-captive control bats differed in their departure times and foraging bout durations, we fit linear mixed-effect models (LMMs) with type of bat and day as fixed effects and bat as a random intercept. We calculated p-values using Satterthwaite's degrees of freedom method with the R package lmerTest). To compare consistency of onsets and durations, we measure the unadjusted repeatability (intra-class correlation or ICC) for each type of bat. To count how often tagged bats departed together, we inspected cases where bats departed the roost within one minute of each other.**

***Preferred associations during foraging***

**To test if repeated foraging encounters occurred among the same bats more than expected by chance, we used a permutation test that compared observed and expected social differentiation while controlling for overlap in foraging times. For social differentiation, we used the coefficient of variation in co-foraging rates, which increases when some pairs have more repeated encounters than others and decreases when pairs have similar encounter rates. We first used a simple and conservative measure of co-foraging: counting the presence or absence of an encounter during each hour outside the roost over 9 days. These counts varied from 0 to 15. If two bats met twice in the same hour, this is still one encounter. We used this method because all bats were sampled evenly within each night and most foraging encounters were brief (median = 1 second). These present vs. absent observations in each hour were swapped to randomize the data. Specifically, we permuted one bat in every dyad to a random possible partner that was also outside the roost during that same day and hour. By randomizing the data this way 5000 times, we generated a null distribution of social differentiation values expected by chance.”**

Line 325:
Out of interest, why do you think they wait so long before departing the roost site after dark?

**We don’t know for sure, but it might have to do with moonlight.**

Lines 326-329:
Is this a possible carry-over effect of captivity?

**Yes, the difference between the previously captive and control group probably is an effect of captivity, but we leave it for the reader to decide for themselves.**

Line 330:
Should moon phase be controlled for in your analyses?

**The effect of the moon is the same for all bats (test and control group). Measuring the effect of moonlight on departure times is outside the scope of our paper which focuses on social behavior.**

Lines 353-357:
Yes, greater than expected by chance. But what is the cattle had been highly clustered for various reasons, meaning the food source is highly concentrated (this is post cattle corralling). Is this just a product of food distribution?

**This scenario could be an explanation if we were comparing the mean encounter rate to a random model, but we were comparing the standardized variance (coefficient of variation) in encounter rates across dyads. In other words, some pairs had consistently high encounter rates whereas other pairs had consistently low encounter rates. These repeated encounters occurred at different times of the night. This high rate of social differentiation cannot be explained by food distribution.**

**For a previous comment, we already revised this text and the corresponding methods section for clarity (Lines 233-256).**

Lines 378-386:
These are quite confusing for the reader to follow. Could you possibly elaborate here and provide more information and detail.

**We agree. We revised this text for clarity.**

**Original text:**

**“Within-roost associations also predicted a greater number of nights with foraging encounters (associations: QAP, β = 0.07, p < 0.001; close-contact association: QAP, β = 0.04, p = 0.021). The relationship between the within-roost association network and the corresponding night’s foraging network was clear within only one of the 8 days (Table S1), but the paired relationships between day and night networks tended to be greater than zero overall (associations: mean β = 0.026, 95% CI = 0.004 to 0.051; close-contact associations: mean β = 0.018, 95% CI = 0.003 to 0.04).**

***Roosting degree centrality predicted foraging degree centrality***

**Bats that were connected to more associates in within-roost association networks also tended to have more associates in the nightly foraging networks”**

**Revised text:**

**“Within-roost associations also predicted a greater number of nights with foraging encounters (associations: QAP, β = 0.07, p < 0.001; close-contact association: QAP, β = 0.04, p = 0.021). When we tested the effect of each pair’s daytime roosting proximity on their foraging encounters on that subsequent night, we found a clear effect within only one of the 8 days (Table S1), but the overall effect size across days was greater than zero (associations: mean β = 0.026, 95% CI = 0.004 to 0.051; close-contact associations: mean β = 0.018, 95% CI = 0.003 to 0.04).**

***Roosting degree centrality predicted foraging degree centrality***

**Bats that associated with more partners within the roost also associated with more partners at night outside the roost (associations: β = 0.034, n = 48 bats, one-tailed p = 0.008; close-contact: β = 0.055, one-tailed p = 0.078; Figure S4).”**

Line 386-388:
"Tended". It's either significant or it is not. It is not.

**This word “tended” was deleted in the revision above. The effect size in this case is actually statistically significant because the 95% CI did not overlap with zero. We note that significance is a slippery concept that depends on how one controls for multiple testing, etc. We therefore avoid the term “significance” and simply present the effect sizes, confidence intervals, and p-values as evidence for the skeptical reader to evaluate in context.**

Line 405:
This reads a little like an afterthought and add on. I would actually suggest removing this entirely from the paper, as it gets lost amongst all the other findings. It would work better as a stand-alone paper, presenting the novelty of this call.

**We have now carefully thought this over. Following the suggestion of the editor (see above), we finally decided to keep this result in the paper and to promote the former supplementary figure on call sonograms to a main text figure.**

Discussion:

Figure 1 does a great job of visualising the logic behind these analyses and findings. I would suggest referring back to it to in the Discussion to keep the same structure and framework.

**Good idea! We now refer back to Figure 1 at the start of the discussion when discussing our predictions and results.**

Line 427:
What would be the benefit of foraging together? It would only be beneficial if individuals co-operate? Otherwise greater risk of prey becoming alert to presence?

**Foraging benefits could be gained by two or more foragers through purely self-interested behavior (i.e. byproduct mutualism), or only one bat could gain a benefit from another (e.g. producer-scrounger dynamics). For instance, bats could feed from an open wound sequentially, or two bats might be better at making a wound than one bat alone, just as two predators can be more successful at biting a prey animal than either alone. We decided not to speculate further here since we lack the data to evaluate these ideas. Instead, our main goal was simply to show multiple lines of evidence that bats reunite with preferred socially bonded partners during foraging.**

**At the beginning of the discussion, line 481, we now refer to the paragraph ‘Implications for cooperation’ (we added the text: “see ‘Implications for cooperation’ “) to point to the following revised text:**

**“Vampire bats might benefit from foraging with socially tolerant partners (rather than alone or with random strangers) by acquiring social information on where to feed or by gaining access to open wounds. A single open wound can sequentially feed several bats, and allowing a close social partner to sequentially feed on the same open wound could be less costly to a successful forager than regurgitating blood to that individual later at the roost.”**

Lines 462-466:
Is it just site preference that explains these findings? Certain individuals have preferred foraging areas, due to previous success there, and thus return?

**It is possible that nonrandomly high rates of foraging encounters are caused by shared site preferences. However, shared site preferences by itself could not explain other findings, such as why bats that shared food more in captivity for the previous 21 months have more similar foraging site preferences. Also, keep in mind that the cows move around and graze at night (as in the provided videos) and they also don’t sleep in the same spot each night. The behavior of the cattle would, therefore, speak against a mere site preference, too. The most parsimonious explanation is that social preferences within the roost extend to outside the roost.**

**We added the sentence to the first paragraph of the discussion:**

**“Taken together, these observations of social foraging cannot be fully explained by non-social factors such as shared site preferences.”**

Line 469:
Over distance can these calls be heard? How close does an individual have to get to another individual to be able to hear these calls?

**We don’t know but we hope to gain more insights in future studies. Answering this question properly would require measuring call intensity with calibrated microphones under standard conditions of temperature and pressure and combining these data with audiograms of frequency sensitivity. However, the fact that bats appeared to exchange calls in the field (as described in the supplement) suggests they could hear each other. Anecdotally, we have seen vampire bats responding to social calls at distances of >10 m.**

Discussion General Comments:

The Discussion does a good job of describing the results, but I don't think it really sets the results into any perspective, and is very narrow focused. It almost exclusively talks about the current study, with very little reference to prior work or work outside the immediate system. The limited reference to prior work is all bats. In contrast, the Introduction is broader and encompassing. As a consequence, the Introduction and Discussion feel quite disconnected.

**We agree with the reviewer that the paragraph ‘*Affiliative and competitive interactions*‘ would benefit from discussing work that had been introduced in the beginning of the manuscript. We added discussion of relevant work on birds, primates, and dolphins highlighting how social relationships may provide access to food or, reversely, how foraging decision may shape social relationships. That is, social bonds may be reinforced by affiliative interactions in different behavioral contexts.**

**We added the following text to this paragraph to broaden the scope and to make the connection to the introduction:**

**“Foraging behavior and social preferences may create a feedback loop. Social relationships can guide foraging decisions and help individuals gain access to defendable food (Borgeaud and Bshary, 2015; Firth et al., 2015). For instance, experimental manipulation of social structure in zebra finches can impact how individuals forage together (Maldonado-Chaparro et al., 2018). Conversely, decisions about where to forage may influence the formation of social bonds. For instance, dolphins that share individual preferences for foraging sites or behaviors are more likely to associate in other contexts (Machado et al., 2019; Methion and Díaz López, 2020). In vampire bats, stable isotope analyses suggest that individuals within the same colony have individualized foraging preferences; they repeatedly target different kinds of prey such as cattle versus sea lions (Streicker and Allgeier, 2016). As in dolphins, shared foraging preferences might similarly help drive social associations in vampire bats.”**

**Please note that the paragraph ‘Implications for cooperation’ has already a quite broad focus discussing the concept of interdependence and the spatial scale of competition. In this paragraph, we cite work on ravens, humans, and group-living animals in general.**

How do individuals recognise each other? It would be useful to have a few sentences describing this, for the benefit of non-specialist readers.

**We agree. We added findings from an earlier study on individual recognition in vampire bats. We revised the text to read:**

**“The ways that specific bats reunited with preferred associates therefore remains unknown, but the downward sweeping calls that we recorded in foraging bats (Figure S6), are similar to individually variable contact calls that vampire bats use to find and recognize preferred partners (Carter and Wilkinson, 2016).”**

I think the Latin names of species need adding where appropriate.

**We defer to the editor and journal style. Given the general non-specialist audience for this paper, we decided to cite the reference which provides the scientific name of the species we mention. For example, saying “the omnivorous greater spear-nosed bat in Trinidad” communicates our main message which is “another species of bat that is not a vampire in a different location” and full taxonomic details are in the reference we cite.**

**We thank this reviewer for these comments which greatly improved the clarity of our writing.**


Reviewer #3:

I very much enjoyed reading this manuscript, and I felt it fit the format of a 'Short Report' perfectly. Specifically, the research uses a range of new technologies and a charismatic system to shed new light on the relationship between social foraging and social relationships (particularly cooperative ones). As well as the broader implications, the findings also provide some novel new natural history type discoveries on this particular species too (which is great). I found that the writing was strong and concise, and that the methods and analyses were intuitive and suitable for providing these findings and the emerging conclusions drawn from them. As such, I only have minor comments on this manuscript (primarily just in relation to clarifying specific aspects of the report).

**Thank you! We are pleased to hear that you enjoyed the manuscript!**

Abstract

Line 30: Change "controlling" to "accounting"

**Fixed**

Line 31: The "and a previously undescribed call type" comes across as a bit 'out-of-the-blue' here. I think if readers first read the abstract before the full manuscript, they won't know what this means or what it is referring to at this point (I didn't).

**We agree. We changed the text as follows:**

**Original text: “Foraging bats demonstrated both affiliative and competitive interactions and a previously undescribed call type”**

**Revised text: “Foraging bats demonstrated both affiliative and competitive interactions with different social calls linked to each interaction type.”**

Introduction

I very much enjoyed the introduction and found it to be broadly appealing and span a great deal of systems. In some places, it felt like it could perhaps benefit on also making reference to other types of benefits/resources (rather than focussing on foraging), as actually a lot of the concepts here apply to any resource or need. For instance, Fleischmann et al. 2011 Current Biology (<https://www.cell.com/current-biology/pdf/S0960-9822(13)00784-7.pdf>) may be of interest to make reference to, as it considers how bats weigh up social relationships compared to access to preferred roosting sites (instead of focussing entirely on foraging). If the authors hope to keep the focus on foraging needs in the introduction (particularly given the nature of a 'short report' format), then perhaps it may just be beneficial to make some reference in the discussion to say that the concepts considered here actually extend beyond foraging, and future work could also be considered in relation to other needs/resources.

**To fit the short report format, we did intend to keep the focus on foraging here given that we have written a number of papers on other aspects and benefits of vampire bat cooperative relationships, and it is the focus on social foraging that is our novel contribution in this paper. However, we have developed and clarified some of the general themes at the end of the introduction and discussion (see below).**

Line 102: I don't think the phrase 'we used unpublished data' is needed here, as (1) I think the data will be published alongside the manuscript? And (2) I don't think previously-unpublished data should be viewed as innately more valuable than published/open data.

**Agreed. We deleted this phrase.**

Line 106-107: The final line of the introduction phrases the study a little too narrow and confined to a single social consideration within a single species, when actually I think the importance of these findings can be phrased in a much broader sense, as they give new general/fundamental insights into this phenomena in animal systems.

**We agree. The revised text now reads: “Multiple lines of evidence show that cooperative relationships in vampire bats extend outside the roost. More generally, our findings illustrate how social relationships and networks can both extend and vary across contexts. “**

Methods

Line 150-151: The MS states
"The results of this constrained permutation test and the unconstrained Mantel test were similar and gave the same conclusion so we report only the results from the double permutation test"
But, given the amount of interest in these analytical techniques at the moment, and how standard models compare to those of null models, I would recommend including both sets of results (and placing one set in the supporting information for the more interested reader to refer to).

**We agree that there is considerable interest in various permutation procedures, although in this case neither of the methods detected an effect. However, we deleted this text in the methods section and now include both sets of results alongside in the supplement (Table S2).** **We renumbered supplemental Tables following the order they are mentioned in the text.**

Line 153: Why was a '1 hour' window chosen here? Were any other thresholds tried?

**We used this arbitrary time binning threshold for reasons of computational time (for ease of reproducibility, we did not want our analysis to take several days), and because we used this threshold in earlier manuscripts. To maintain the comparability of our analyses across different studies, we often use the same thresholds for time bins and signal intensity. We did not use other (unreported) thresholds.**

Line 189-190: Change "see figure 2 in Ripperger et al. (2020b)" to "see Ripperger et al. (2020b)"

**Fixed**

Results

Through the results, I'm unsure about which type of p values are being reported. Are these all the p values associated with the standard model parameters that are being reported? Or are these the p values derived from a separate permutation test against the standard model? If it is the latter, I recommend referring to it as 'P-null=' instead of just 'P='. If it is the former, then I think this needs specifying right at the start (and made clear in the methods).

**We now clarify this aspect of our data analyses. We now use the term “p-null” and at the end of the methods, we now say** **“Throughout the results we use ‘p-null’ to indicate p-values that come from a null model where permutations were constrained within day.” Thanks for bringing this to our attention.**

Line 356: The wording of '10 among 8' is a little confusing (sounds impossible at first notice), and this comes from this difficulty in the separation between foraging encounters and foraging pairs. I think this sentence and related phrases needs rephrasing for clarity. Perhaps it needs to be that each time a number is used, the associated term is also used e.g. instead of just saying '…10…', say '….10 foraging encounters…', etc.

**This indeed seems confusing. We changed the text as follows:**

**Original text: “Most of these foraging encounters occurred at locations outside our sampled areas, but 10 (among eight pairs) occurred near the other base stations…”**

**Revised text: “Most of these foraging encounters occurred at locations outside our sampled areas, but 10 encounters (involving 8 pairs of bats) occurred near the other base stations...”**

L388: Why were one-tailed tests used here? Perhaps I missed this explanation in the methods, but would be good to make clear in results too briefly if using these.

**We agree that we should explain this better. We added the following text immediately after the test results:**

**“P-values are one-tailed because the center of the expected β values from the null model was not zero (Figure S4).”**

**Here is Figure S4 below. We could estimate a two-tailed p-value (by doubling the one-sided p-value or measuring the proportion of expected values that are as distant from the mean or median as the observed, but these estimates are only valid if the distribution is symmetrical. In contrast the one-sided p-value is correct and straightforward to interpret without this assumption. Also, one-sided p-values make sense here, because there is no a priori prediction for a negative slope.**

**Figure S4. Foraging degree centrality is predicted by roosting degree centrality.** Blue histograms show expected coefficients for the same model fit to randomized data generated by our null model. Red lines show the observed coefficients. Subtitles provide the observed coefficient, the 95% quantiles for the expected coefficients, the one-sided p-value, and number of permutations. Proximity of about 50 cm is required for “roosting associations” and about 2 cm proximity is needed for “close contact”, so close-contact networks are sparser.

Discussion

Line 423-425: This second sentence of the discussion contains the key findings, but is currently a little complex to digest easily. I suggest breaking this into two sentences that follow on from one another.

**Agreed. Fixed.**

Line 483-503: I think that these two short (and separately sub-headed) paragraphs would be better if they were integrated into a single paragraph/subheading (as social dominance is captured broadly within competition anyway).

**We removed the sub-heading “Implications for social dominance”.**

Line 540-541: The final sentence of the discussion is phrased a little too methodological for an paper with such important fundamental findings. I think it would be more exciting to end the discussion with a sentence or so about the breadth of these findings and what they now suggest should be the future priorities/possibilities of interest.

**We agree. We revised this sentence following a previous comment. It now reads:**

**“Rapid standardized high-throughput methods for measuring social network structure, such as social proximity sensors, allow for social networks to be mapped quickly across multiple populations and species, enabling comparative studies investigating evolutionary and ecological drivers of social complexity across species.”**

Figure 1
Really found this figure very helpful to see exactly what was being tested in the manuscript. I suggest slightly changing the figure title from "…how within-roost relationships predict foraging…" to "…how within-roost relationships could predict foraging…"

**Thanks! Done.**

Figure 2
Another great and useful figure. One question though, the legend states "To facilitate visual comparisons, we fixed the spatial coordinates of each node" but unclear how it was fixed? How were the positions in the circles defined initially, and which network were the positions fixed on? Or was it a 'best fit' position taken from average position over two separate networks?

**We provide the code for making these plots in the Figshare repository, and we added this text to the Figure caption:**

**“To facilitate visual comparisons, we applied the Fruchterman-Reingold algorithm to a fully connected unweighted network and used this layout to fix the spatial coordinates of nodes across networks (except for the sparse night-by-night foraging networks).”**

I hope the authors find these minor comments useful for this interesting work.
Best wishes
Josh Firth (please note, I sign all of my reviews).

**Thanks Josh for your kind and constructive comments!**

**Other changes:**

**In our original paper, we report two kinship analyses that were actually redundant. The first model is better because it treats kinship as a continuous variable and controls for foraging bout overlaps, whereas the second model (which we did based on a question by a reader of the preprint) labels pairs categorically as related (r>0.1) vs unrelated (r<0.1). We therefore deleted the second model, and instead, we used these categories to give a simple and intuitive idea of the effect of kinship by simply providing the median durations for kin and nonkin pairs (i.e. 9 seconds vs 1 second). The revised text now reads:**

**“Kinship predicted the number of nights with foraging encounters (QAP, β = 15.4, n = 46 bats, p-null < 0.0001) and foraging encounter time (β = 15.4, n = 47 bats, p-null = 0.022) even when controlling for bout overlap (MRQAP, β = 0.10, p-null = 0.002). The median duration of a foraging encounter for close kin (kinship > 0.1) was 9 s, compared to 1 s for nonkin (kinship < 0.1).”**

**We have updated the scripts and files on Figshare, so that the analyses are ordered in the order they should be run.**
